# Clinical Features of Embolic Stroke of Undetermined Source

**DOI:** 10.3389/fneur.2020.00058

**Published:** 2020-02-05

**Authors:** Weijing Wang, Xiaomei Tang, Wei Liu, Ke Jia, Xingquan Zhao, Fengchun Yu

**Affiliations:** ^1^Department of Neurology, Beijing Haidian Hospital, Beijing Haidian Section of Peking University Third Hospital, Beijing, China; ^2^Department of Neurology, Haidian Section of Peking University Third Hospital, Beijing, China; ^3^Department of Neurology, Beijing Tiantan Hospital, Capital Medical University, Beijing, China; ^4^China National Clinical Research Center for Neurological Diseases, Beijing Tiantan Hospital, Capital Medical University, Beijing, China; ^5^Center of Stroke, Beijing Institute for Brain Disorders, Beijing, China

**Keywords:** embolic stroke of undetermined source, acute ischemic stroke, prolonged heart-rhythm monitoring, Cardiogenic embolism, large-artery atherosclerosis, small-artery occlusion lacunar

## Abstract

**Background and Objective:** One-third of ischemic strokes have no identifiable cause following standard evaluation. In 2014, researchers have proposed the concept of Embolic Stroke of Undetermined Source (ESUS). The purpose of this study was to report the clinical characteristics of ESUS and its difference from cardiogenic embolism (CE), large-artery atherosclerosis (LA), and small-artery occlusion lacunar (SA).

**Methods:** Acute ischemic stroke (AIS) patients admitted to the department of Beijing Haidian Hospital from January 2017 to December 2017 were prospectively and consecutively enrolled. Base-line characteristics were collected. Stroke etiologies were presented and compared. We compared the clinical features and infarct sites of patients with acute cerebral infarction of different etiologies.

**Results:** A total of 119 AIS patients were analyzed in the study. There were 33 (27.73%) cases in ESUS group, 11 (9.24%) cases in CE group, 45 (37.82%) cases in LAA group and 30 (25.21%) cases in SA group. There were significant differences between the ESUS group and the CE group in the NIHSS score [3 (1.5–5) vs. 6 (2–20), *p* = 0.007], Modified Rankin Score [19, (57.58) vs. 9, (81.82), *p* = 0.008], hemorrhagic transformation [0, (0) vs. 5, (45.45), *p* < 0.001], and left atrial diameter [37.09 ± 3.16 vs. 41.73 ± 5.00, *p* = 0.001]. ESUS group and LA group have different mRS scores [19, (57.58) vs. 42, (93.33), *p* < 0.001]. ESUS group and SA group have different mRS scores [19, (57.58) vs. 28, (93.33), *p* = 0.001]. During 1 year follow-up, there were 5 cases (15.15%) in ESUS group, 3 cases (27.27%) in CE group, 3 cases (6.67%) in LA group, and 1 case (3.33%) in SA group with ischemic stroke (cerebral infarction or transient ischemic attack).

**Conclusion:** ESUS is more similar to atherosclerotic cerebral infarction in clinical features, but the distribution of lesions is more similar to cardiogenic embolism, suggesting that the pathogenesis of ESUS needs to be further explored.

## Introduction

In 2014, the Cryptogenic Stroke/ESUS International Working Group first proposed the concept of embolic stroke of undetermined source (ESUS) (1) as a new subtype of stroke. ESUS refers to non-lumen infarct ischemic stroke excluding intracranial and extra cranial vascular stenosis and definite source of cardiogenic emboli (1). Researchers termed these ESUS and argued that this entity would respond to anticoagulation. Two recent randomized clinical trials (2, 3) have not upheld this hypothesis, leading to questions about the ESUS concept. This study focused on the clinical features and infarct site characteristics of ESUS in order to further study of the pathophysiological mechanism.

## Materials and Methods

### Study Population

We prospectively and consecutively enrolled AIS patients admitting to the Department of Neurology of Beijing Haidian Hospital from January 2017 to December 2017. There are 3.48 million people and 16 general hospitals in Haidian District of Beijing. Beijing Haidian Hospital is one of them. Intravenous thrombolysis can be given if the patient arrives in the emergency within 4.5 h and meets the criteria of the Chinese guidelines for the treatment of acute cerebrovascular disease. This study is an observational study, without intervention in the treatment.

Eligibility Criteria: (1) Age 18 years or older; (2) Acute ischemic stroke diagnosed by imaging within 6 weeks (MRI completed within 2 weeks after onset, other examinations were completed within 6 weeks after the onset of the disease); (3) Necessary examinations for etiological diagnosis after stroke: 12-lead ECG, 24-h HOLTER, cranial MRI, CTA or MRA of head and neck vessels, transthoracic/esophageal echocardiography, blood routine, coagulation function and thrombosis-prone screening; (4) Informed consent.

Exclusion criteria: (1) intracranial hemorrhagic diseases or tumors, infections, etc. (2) non-primary ischemic stroke; (3) other causes of cerebral infarction (such as hypercoagulability, tumors, etc.); (4) severe organ dysfunction, such as liver and kidney dysfunction; and (5) severe stroke patients who are expected to survive for <1 year. This study was conducted in accordance with the principles of the Helsinki Declaration, approved by the Ethics Committee of Beijing Haidian Hospital and supported by the Youth Project Foundation of Haidian Hospital (Project No. KYQ2017005). All participants received written informed consent.

**Figure 1:**
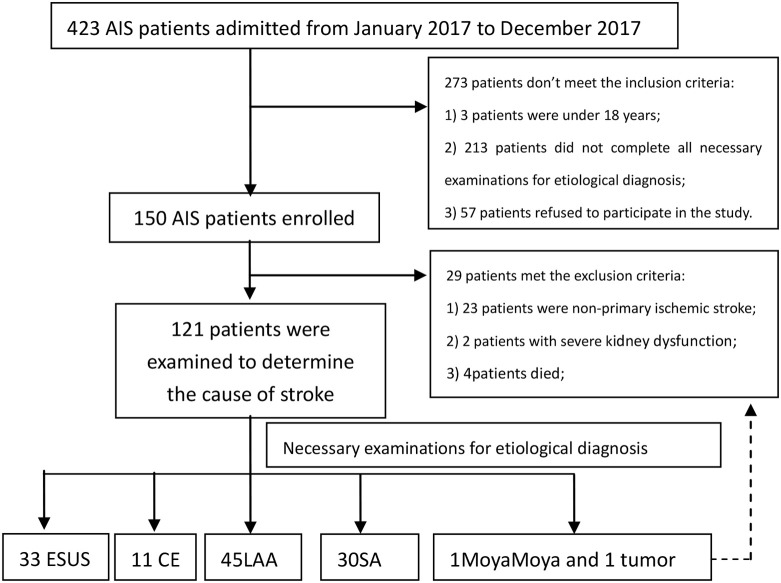
Flow diagram of study enrollment.

A total of 119 AIS patients were analyzed in the study. There were 33 (27.73%) cases in ESUS group, 11 (9.24%) cases in CE group, 45 (37.82%) cases in LAA group and 30 (25.21%) cases in SA group (Figure 1).

### Data Collection

Patient demographic characteristics and baseline National Institutes of Health Stroke Scale (NIHSS) score were collected on admission. Clinical and laboratory information was also collected, including hypertension, diabetes, atrial fibrillation (AF), smoking, total cholesterol, triglyceride, high density lipoprotein (HDL), low-density lipoprotein (LDL). Patient blood samples were collected within 24 h of admission. Diagnosis of hypertension and diabetes mellitus were defined as having an evident history of disease from interviewing the patient or diagnosis during the current treatment in hospital. AF was defined as having a history of persistent or paroxysmal AF, based on previous electrocardiograms or prolonged heart-rhythm monitoring during hospitalization. Imaging data, including head CT /MRI, MRA and CT angiography, were interpreted by experienced radiologists. The infarct location and hemorrhagic transformation were recorded. We registered the secondary preventive drug regimen at discharge. After discharge, the patients were followed up by telephone on the 30th day of onset, and the modified Rankin Score (mRS) on the 30th day of onset were recorded. The patients were followed up by telephone for 1 year to record the recurrence of stroke within 1 year.

### Stroke Classification

Diagnostic criteria for different types of stroke: Diagnostic criteria for stroke types are TOAST classification proposed by Adams et al. (4). They are diagnosed as large-artery atherosclerosis (LA), cardiogenic embolism (CE), small-artery occlusion lacunar (SA), other etiological types (ODE) and cryptogenic stroke (CS). Criteria for diagnosis of ESUS (1): (1) Stroke detected by CT or MRI that is not lacunar, (2) Absence of extra cranial or intracranial atherosclerosis, (3) causing ≥50% luminal stenosis in arteries supplying the area of ischemia, (4) No major-risk cardio embolic source of embolism, (5) No other specific cause of stroke identified (e.g., arteritis, dissection, migraine/vasospasm, drug misuse).

### Patients Follow-Up

At 90 days and 1 year after AIS onset, the recurrence information of all patients was assessed through telephone follow-up interview to obtain information on recurrence. Every AIS patients recruited left at least two phone numbers. For patients who did not follow-up, we conducted a telephone follow-up interview once a week for on three instances. Telephone follow-up was centralized for all included patients and utilized a standardized interview protocol. The interviewers were trained on the interview protocol.

### Statistical Analysis

SPSS 16.0 statistical software was used to analyze the data. Continual variables were given as mean and SD, and categorical variables were calculated as percentages. Independent sample *t*-test was used for continuous variables. Categorical variables were examined by_2 test. Bilateral test was used for all the analyses, *P* < 0.05 showed that the difference was significant.

## Results

### Study Participants and Baseline Characteristics

A total of 119 patients with Acute ischemic stroke were included in this study. According to the etiology of ischemic stroke, the patients were divided into four groups: ESUS group, CE group, LAA group, SA occlusion group. All ESUS patients underwent long-term ECG monitoring (72 h). Two of them were confirmed to be cryptogenic AF and were included in CE group. All ESUS patients completed screening for deep venous thrombosis and patent foramen ovale (PFO) to prove that they did not have cardiogenic embolism caused by PFO. After the screening process, 33 cases were still in accordance with the ESUS diagnosis, accounting for 27.73% of all types of stroke in our research center. There were 11 (9.24%) cases in CE group, 45 (37.82%) cases in LA group, 30 (25.21%) cases in SA group. A total of 119 patients were recruited. The baseline demographic characteristics of the study population, stratified by etiology, are summarized in Table 1.

**Table 1:** The baseline characteristics of the study population.

**Variable**		**Total (*n* = 119)**	**ESUS (*n* = 33)**	**CE (*n* = 11)**	**LA (*n* = 45)**	**SA (*n* = 30)**
Demographics						
Gender male, *n* (%)		84, (70.59)	25, (75.76)	6, (54.55)	34, (75.56)	19, (63.33)
Age, years, mean ± SD		66.39 ± 12.74	69.18 ± 12.27	68.55 ± 14.51	65.27 ± 12.79	64.20 ± 12.48
Medical history						
Smoking, *n* (%)		42, (35.29)	9, (27.27)	2, (18.18)	18, (40.00)	13, (43.33)
Diabetes, *n* (%)		40, (33.61)	8, (24.24)	4, (36.36)	18, (40.00)	10, (33.33)
Hypertension, *n* (%)		71, (59.66)	19, (57.58)	5, (45.45)	32, (71.11)	15, (50.00)
Laboratory						
Total cholesterol, mmol/L, mean ± SD		4.22 ± 1.10	4.34 ± 0.88	4.10 ± 0.97	4.06 ± 1.21	4.38 ± 1.19
Triglyceride, mmol/L, mean ± SD		1.62 ± 0.87	1.41 ± 0.67	1.54 ± 0.72	1.57 ± 0.77	1.97 ± 1.16
High density lipoprotein, mmol/L, mean ± SD		1.18 ± 0.41	1.17 ± 0.26	1.10 ± 0.31	1.12 ± 0.28	1.31 ± 0.67
Low density lipoprotein, mmol/L, mean ± SD		2.743 ± 0.96	2.94 ± 0.91	2.51 ± 0.85	2.60 ± 1.02	2.79 ± 0.95
Fasting blood glucose, mmol/L, mean ± SD		6.79 ± 2.48	6.42 ± 1.99	6.73 ± 1.90	6.93 ± 3.00	7.02 ± 2.33
Clinical investigations						
NIHSS score, median (IQR)		2 (1–5)	3 (1.5–5)	6 (2–20)	2 (1–4)	1.5 (1–4)
Modified rankin score, ≤ 1, *n* (%)		98, (82.35)	19, (57.58)	9, (81.82)	42, (93.33)	28, (93.33)
Area of infarction	Anterior circulation, *n* (%)	73, (61.34)	19, (57.58)	8, (72.73)	28, (62.22)	18, (60.00)
	Posterior circulation, *n* (%)	38, (31.93)	10, (30.30)	2, (18.18)	15, (33.33)	11, (36.67)
	Both circulation, *n* (%)	8, (6.72)	4, (12.12)	1, (9.09)	2, (4.44)	1, (3.33)
	Bilateral, *n* (%)	13, (10.92)	7, (21.21)	1, (9.09)	2, (4.44)	3, (10.00)
Hemorrhagic transformation, *n* (%)		9, (7.56)	0, (0)	5, (45.45)	3, (6.67)	1, (3.33)

*ESUS, Embolic Stroke of Undetermined Source; CE, cardiogenic embolism; LA, large-artery atherosclerosis; SA, small-artery occlusion lacunar*.

### The Baseline Characteristics Between Groups

Baseline characteristics were compared between groups. There were no differences in gender, age, history of smoking, history of diabetes, history of hypertension, total cholesterol, high density lipoprotein, low density lipoprotein, and fasting blood glucose among the groups (Table 2).

**Table 2:** The baseline demographic characteristics between ESUS and other etiologies.

**Variable**	**ESUS (*n* = 33)**	**CE (*n* = 11)**	***P***	**ESUS (*n* = 33)**	**LA (*n* = 45)**	***p***	**ESUS (*n* = 33)**	**SA (*n* = 30)**	***p***
Gender male, *n* (%)	25, (75.76)	6, (54.55)	0.256	25, (75.76)	34, (75.56)	0.984	25, (75.76)	19, (63.33)	0.283
Age, years, mean ± SD	69.18 ± 12.27	68.55 ± 14.51	0.887	69.18 ± 12.27	65.27 ± 12.79	0.178	69.18 ± 12.27	64.20 ± 12.48	0.116
Smoking, *n* (%)	9, (27.27)	2, (18.18)	0.701	9, (27.27)	18, (40.00)	0.243	9, (27.27)	13, (43.33)	0.182
Diabetes, *n* (%)	8, (24.24)	4, (36.36)	0.457	8, (24.24)	18, (40.00)	0.145	8, (24.24)	10, (33.33)	0.425
Hypertension, *n* (%)	19, (57.58)	5, (45.45)	0.509	19, (57.58)	32, (71.11)	0.214	19, (57.58)	15, (50.00)	0.547
Total cholesterol (mmol/L)	4.34 ± 0.88	4.10 ± 0.97	0.441	4.34 ± 0.88	4.06 ± 1.21	0.258	4.34 ± 0.88	4.38 ± 1.19	0.882
Triglyceride (mmol/L)	1.41 ± 0.67	1.54 ± 0.72	0.591	1.41 ± 0.67	1.57 ± 0.77	0.344	1.41 ± 0.67	1.97 ± 1.16	0.021^*^
High density lipoprotein (mmol/L)	1.17 ± 0.26	1.10 ± 0.31	0.436	1.17 ± 0.26	1.12 ± 0.28	0.425	1.17 ± 0.26	1.31 ± 0.67	0.285
Low density lipoprotein (mmol/L)	2.94 ± 0.91	2.51 ± 0.85	0.175	2.94 ± 0.91	2.60 ± 1.02	0.126	2.94 ± 0.91	2.79 ± 0.95	0.521
Fasting blood glucose (mmol/L)	6.42 ± 1.99	6.73 ± 1.90	0.654	6.42 ± 1.99	6.93 ± 3.00	0.398	6.42 ± 1.99	7.02 ± 2.33	0.280
NIHSS score	3 (1.5–5)	6 (2–20)	0.007^**^	3 (1.5–5)	2 (1–4)	0.188	3 (1.5–5)	1.5 (1–4)	0.058
Modified rankin score, ≤ 1, *n* (%)	19, (57.58)	9, (81.82)	0.008^**^	19, (57.58)	42, (93.33)	<0.001^**^	19, (57.58)	28, (93.33)	0.001^**^
Hemorrhagic transformation (*n*,%)	0, (0)	5, (45.45)	<0.001^**^	0, (0)	3, (6.67)	0.130	0, (0)	1, (3.33)	0.290
Left atrial diameter (mm)	37.09 ± 3.156	41.73 ± 5.00	0.001^**^	37.09 ± 3.156	36.60 ± 1.156	0.340	37.09 ± 3.156	36.23 ± 1.135	0.164

*ESUS, Embolic Stroke of Undetermined Source; CE, cardiogenic embolism; LA, large-artery atherosclerosis; SA, small-artery occlusion lacunar*.

* p <0.05;

** *p <0.01*.

There were significant differences between the ESUS group and the CE group in the NIHSS score [3 (1.5–5) vs. 6 (2–20), *p* = 0.007], Modified Rankin Score [19, (57.58) vs. 9, (81.82), *p* = 0.008], hemorrhagic transformation [0, (0) vs. 5, (45.45), *p* < 0.001], and left atrial diameter [37.09 ± 3.16 vs. 41.73 ± 5.00, *p* = 0.001]. ESUS group and LA group have different mRS scores [19, (57.58) vs. 42, (93.33), *p* < 0.001]. ESUS group and SA group have different mRS scores [19, (57.58) vs. 28, (93.33), *p* = 0.001].

### Infarct Site in Different Groups

The Infarct Sites were compared between groups (Table 3). There was no difference in the infarct site between the ESUS group and the CE group [4, (12.12) vs. 1, (9.09), *p* = 0.784; 7, (21.21) vs. 1, (9.09), *p* = 0.367]; ESUS had more bilateral infarcts than the LA group [7, (21.21) vs. 2, (4.44), *p* = 0.022], and ESUS involved more anterior and posterior circulation than SA group [4, (12.12) vs. 0, (0.00), *p* = 0.026].

**Table 3:** Difference of infarct location between groups.

**Infarct location**	**ESUS (*n* = 33)**	**CE (*n* = 11)**	***P***	**ESUS (*n* = 33)**	**LA (*n* = 45)**	***P***	**ESUS (*n* = 33)**	**SA (*n* = 30)**	***P***
Both (anterior and posterior) circulation, *n* (%)	4, (12.12)	1, (9.09)	0.784	4, (12.12)	2, (4.44)	0.209	4, (12.12)	0, (0.00)	0.026^*^
Bilateral, *n* (%)	7, (21.21)	1, (9.09)	0.367	7, (21.21)	2, (4.44)	0.022^*^	7, (21.21)	3, (10.00)	0.224

*ESUS, Embolic Stroke of Undetermined Source; CE, cardiogenic embolism; LA, large-artery atherosclerosis; SA, small-artery occlusion lacunar*.

* *p <0.05*.

### Treatment

All patients were given a secondary prevention treatment for cerebrovascular disease after discharge. For the treatment of ESUS, the choice of anticoagulation or antiplatelet is still inconclusive, so 31 patients received a single antiplatelet treatment. One received aspirin and clopidogrel antiplatelet therapy because of recent femoral stenting. One used anticoagulation because of recent pulmonary embolism. Eight patients in the CE group were given anticoagulant therapy according to the Chinese Guidelines for Secondary Preventive Therapy of Cerebrovascular Disease 2014. Two patients were given dual antiplatelet therapy, one of which was due to recent coronary stenting and the other was due to a high risk of bleeding, one patient received clopidogrel only because of high bleeding risk. Patients in the LA and SA groups received antiplatelet therapy according to the Chinese Cerebrovascular Disease Treatment Guidelines 2014.

### Clinical Prognosis

**Figure 2:**
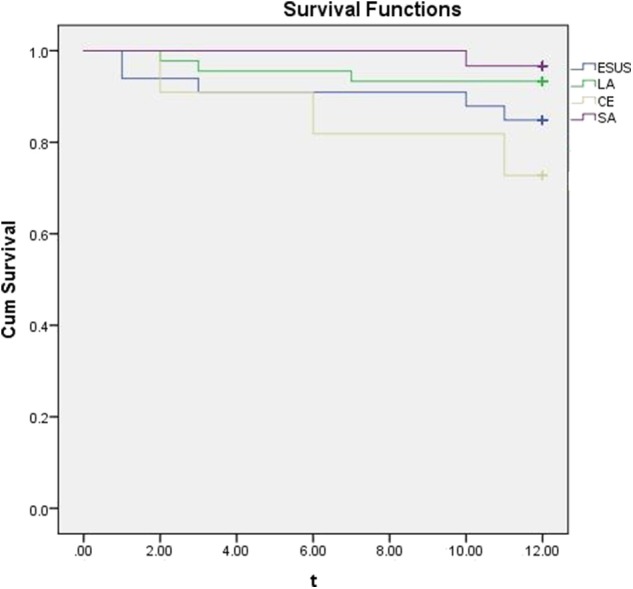
A Kaplan-Meier analysis with different etiologies of stroke.

During the 1 year following, there were 5 cases (15.15%) in ESUS group, 3 cases (27.27%) in CE group 3 cases (6.67%) in LA group and 1 case(3.33%) in SA group with ischemic stroke (cerebral infarction or transient ischemic attack). The results was presented by a Kaplan-Meier analysis (Figure 2).

## Discussion

Stroke accounts for 10% of the world's deaths and causes severe long-term disabilities (5). At present, AIS is usually classified according to TOAST classification, and the corresponding secondary prevention and treatment plan is given according to the etiology. However, about one third of AIS has no obvious reason after standardized evaluation (6, 7). In 2014, Hart et al. (1) put forward the concept of ESUS, and then a series of international studies on the possible mechanism, natural history, and secondary prevention of ESUS were carried out.

Ntaios et al. (8) conducted a retrospective study in 2015. 2735 AIS patients between 1992 and 2011 were analyzed. 275 (10%) of them met the ESUS diagnostic criteria. Takasugi et al. (9) retrospectively analyzed 623 cases of AIS, and found 147 cases (23.6%) were in accordance with ESUS diagnosis. In this study, 150 patients were screened strictly, and 33 cases (22%) were diagnosed with ESUS.

ESUS as a new type of stroke accounts for a large proportion of AIS. This concept was proposed solely because Hart et al. (1) believe that as a new subtype of stroke, it is likely to respond to anticoagulation therapy. However, the conclusions of two recent randomized clinical trials (2, 3) do not support this hypothesis. It is doubtful whether the cause of ESUS is not a homogeneous group of causes, but a mixture of multiple causes, which leads to negative results of anticoagulation therapy.

At present, many related scholars have proposed many possible causes of ESUS, which can be roughly divided into two categories: one is the embolism mechanism that may respond to anticoagulation therapy, the other is the embolism mechanism that may not respond to anticoagulation therapy. Embolization mechanisms that respond to anticoagulation therapy include cryptogenic atrial fibrillation (10–12), atrial heart disease (13, 14), unrecognized myocardial infarction (15), patent foramen ovale (16), tumor (17), and so on. Another type of embolization mechanisms that may not respond to anticoagulation therapy include non-stenosis atherosclerosis (18, 19) and non-atherosclerosis. Sclerosing cerebrovascular disease (dissection, infection, etc.).

Because the etiology of ESUS is not yet clear, it may be caused by many of the above reasons, but this study prolonged the duration of ECG monitoring and excluded some embolic stroke caused by subclinical atrial fibrillation. From the distribution of the lesion, it is more similar to embolism formed by embolism from heart or aortic arch, because it involves more left and right bilateral and anterior and posterior circulation. However, unlike simple cardiogenic embolism, this kind of embolism may be smaller, so the severity of clinical neurological impairment in ESUS patients in this study is lower than that in patients with cardiogenic embolism, which provides ideas for the follow-up study.

## Limitations

The sample size of this study is small, and patients with expected survival of <1 year are excluded, so it's possible to underestimate the severity of stroke in each group.

## Data Availability Statement

All datasets generated for this study are included in the article/supplementary material.

## Ethics Statement

The studies involving human participants were reviewed and approved by the Ethics Committee of Beijing Haidian Hospital. The patients/participants provided their written informed consent to participate in this study.

## Author Contributions

WW and FY contributed conception and design of the study. XT and WL organized the database. KJ performed the statistical analysis. WW wrote the first draft of the manuscript. XT wrote sections of the manuscript. XZ revised the manuscript. All authors contributed to manuscript revision, read and approved the submitted version.

### Conflict of Interest

The authors declare that the research was conducted in the absence of any commercial or financial relationships that could be construed as a potential conflict of interest.
